# A Systematic Review of Comparative Effectiveness of Interventions for Low Anterior Resection Syndrome: Impacts on Bowel Function and Quality of Life

**DOI:** 10.7759/cureus.72772

**Published:** 2024-10-31

**Authors:** Mehwish Ansar, Sruthi Boddeti, Khutaija Noor, Aparna Malireddi, Mahlet Abera, Suchith B Suresh, Iana Malasevskaia

**Affiliations:** 1 General Surgery, California Institute of Behavioral Neurosciences & Psychology, Fairfield, USA; 2 General Surgery, Wirral University Teaching Hospital, Wirral, GBR; 3 Medicine, California Institute of Behavioral Neurosciences & Psychology, Fairfield, USA; 4 Foundation of Clinical Research, Harvard Medical School, Boston, USA; 5 Neuropsychiatry, PsychCare Consultant Research, Saint Louis, USA; 6 Internal Medicine, Shadan Institute of Medical Sciences, Peeramcheru, IND; 7 Internal Medicine, California Institute of Behavioral Neurosciences & Psychology, Fairfield, USA; 8 Internal Medicine, Montefiore Medical Center, Wakefield Campus, Newburgh, USA; 9 Obstetrics and Gynecology, Private Clinic 'Yana Alexandr', Sana'a, YEM; 10 Research and Development, California Institute of Behavioral Neurosciences & Psychology, Fairfield, USA

**Keywords:** bowel function, colorectal cancer surgery, lars, low anterior resection, low anterior resection syndrome, quality of life, rectal cancer surgery, sphincter sparing rectal surgery, systematic review

## Abstract

Low anterior resection syndrome (LARS) is a common complication following sphincter-preserving surgical resection for rectal cancer, characterized by symptoms such as fecal incontinence, urgency, and altered bowel habits, which significantly affect patients' quality of life. This cluster of symptoms not only limits their day-to-day physical activity but also has a debilitating effect on their emotional and mental well-being, undermining their integration and overall psychological health. This systematic review aimed to evaluate the effectiveness of invasive and non-invasive interventions for LARS, including pelvic floor rehabilitation, transanal irrigation, and various surgical options such as sacral neuromodulation and stoma formation, either as part of primary surgery or as a definitive treatment option for refractory cases. We systematically searched relevant databases for studies published in the last decade, focusing on adult patients diagnosed with LARS post-low anterior resection (LAR), with outcomes assessed through bowel function and quality of life metrics. The review identified six studies that met our eligibility criteria; a pooled cohort of 794 patients was identified, with sample sizes ranging from 37 to 430 participants. Our analysis revealed that pelvic floor rehabilitation significantly improves bowel function and quality of life in patients with LARS; yet, the optimal management approach remains unclear due to variability in patient responses. These findings highlight the inherent complexity and heterogeneity of LARS management, underscoring the necessity for multifaceted and individualized treatment strategies. Although pelvic floor rehabilitation shows promise, especially among motivated patients, its long-term sustainability remains uncertain. Surgical options are typically reserved for severe cases and carry significant risks and psychological impacts. For patients identified as being at high risk for LARS, treatment options must be considered and discussed at an earlier phase of their care. Our review concludes with the need for a tailored, patient-centered approach to managing LARS, highlighting the importance of ongoing research to fill existing evidence gaps. There is a need for translational research across various treatment modalities, comparing their effects, cost-effectiveness, implementation strategies, and the consequent effects on patients' quality of life and mental health.

## Introduction and background

Colorectal cancer (CRC) is the third most common malignancy and second in mortality globally, making up approximately 10% of oncological cases. Interestingly, the incidence of CRC is declining in the older population and increasing among young adults [1,2].

Due to recent advances in rectal cancer, not only have the oncological outcomes improved, but sphincter-sparing surgery has now become the standard of care for upper- and mid-rectal cancers [3]. Low Anterior Resection (LAR) is the standard oncological surgical procedure involving the removal of the tumor in the rectum while maintaining bowel continuity by performing a colorectal anastomosis. Preventing a stoma positively impacts the patient’s recovery and quality of life. In spite of the nerve and sphincter-saving procedures, 60% to 90% of patients who undergo LAR experience Low Anterior Resection syndrome (LARS), which affects their quality of life (QoL) [4].

According to the International Consensus Definition of LARS, to meet the definition of LARS, a patient must have had an anterior resection (sphincter-preserving rectal resection) and exhibit one of eight symptoms resulting in one of eight consequences to qualify for the diagnosis of LARS [5]. The LARS score is a questionnaire assessing bowel dysregulation following sphincter-sparing surgery (LAR), with each item assigned a designated score. The score ranges from 0 to 42 and is categorized as no LARS (0-20), minor LARS (21-29), and major LARS (30-42) [6].

The impact of LARS on an individual's quality of life extends beyond physical limitations. Patients with LARS often experience unpredictable bowel habits, leading to distress and embarrassment. Despite lifestyle modifications that mitigate physical discomfort, the psychological toll can persist, significantly affecting overall well-being [7]. It is believed that bowel adaptation will occur approximately 18 months after the surgery, but some patients may experience permanent bowel dysfunction [7].

Management of LARS is remarkably challenging for healthcare professionals owing to its multifaceted and complex nature. The complexity of the wide range of symptoms, severity, and unpredictability has made it difficult to standardize a singular treatment modality until now, and treatment has been tailored to each individual’s symptoms. Additionally, the psychological impact of LARS necessitates a comprehensive management approach. To address both the psychological and emotional aspects of the syndrome, an individualized treatment plan and ongoing support are often required [8].

The Preoperative Low Anterior Resection Syndrome Score (POLARS) is a useful online predictive tool for the potential risk of bowel dysfunction and its severity before anterior resection, and it identifies those who need extra support post-operatively [9]. Post-op diagnosis of LARS comprises a combination of patient-reported symptoms and clinical assessment, followed by specific tools such as the LARS score questionnaire [10] to evaluate the severity of the symptoms. Further diagnostic tests, like endoanal ultrasound or anorectal manometry, may be performed to assess rectal and sphincter function [10].

A significant challenge in managing LARS is the lack of a universally effective treatment. At present, treatment modalities include dietary modifications, drug therapy (including loperamide, a 5-HT3 antagonist), rehabilitation of the pelvic floor muscles, and transanal irrigation [11,12]. For cases of refractory major LARS, neuromodulation (sacral or percutaneous tibial nerve stimulation), antegrade enemas, and permanent stoma for fecal diversion may be considered [13,14]. However, these interventions frequently offer limited relief, requiring patients to try different modalities before finding an effective combination.

The aim of this systematic review was to evaluate the comparative effectiveness of various management strategies for LARS in adults following rectal surgery. We assessed interventions including pelvic floor rehabilitation, transanal irrigation, surgical options, and neuromodulation techniques. Key outcomes of interest included improvements in bowel function and QoL. By synthesizing the current evidence, we seek to provide healthcare professionals with actionable insights that can enhance patient care and inform future clinical guidelines for the management of LARS.

## Review

Methodology

This systematic review was conducted following the Preferred Reporting Items for Systematic Reviews and Meta-analyses (PRISMA) 2020 guidelines [15]. The primary research question guiding this review was: In adult patients with LARS, how have pelvic floor rehabilitation and surgical options improved bowel function and QoL?

Eligibility Criteria

An extensive search was conducted to identify manuscripts evaluating different treatment modalities, such as pelvic floor rehabilitation, neuromodulation, and surgical options like stoma formation, as effective treatments for LARS. These studies included patients aged over 18 diagnosed with LARS. Manuscripts published between January 2014 and August 2024 in the English language were included. The outcomes of interest included bowel function, assessed through frequency, consistency, urgency, and incontinence, as well as QoL, measured using analytical tools. In Table 1, we provide a detailed outline of the eligibility criteria for our systematic review.

**Table 1:** Eligibility Criteria for Study LARS: low anterior resection syndrome; RCTs: randomized controlled trials; QoL: quality of life.

Criteria	INCLUSION CRITERIA	EXCLUSION CRITERIA
Participants	Adults diagnosed with LARS following rectal cancer surgery.	Paediatric populations or those with LARS due to diagnoses other than rectal cancer.
Publication Type /Study Design	RCTs, non-randomized controlled trials, Cohort studies, or Case-control studies	Case series or reports, animal studies, abstracts, expert opinion, editorials, non-completed studies, studies without results, commentaries, review articles
Timeframe	Studies published from January 2014 to August 5, 2024	Studies published after search strategy was concluded (August 5, 2024)
Intervention	Studies evaluating management strategies for LARS following rectal cancer surgery	Studies focused on LARS management for other pathologies.
Outcomes	Studies reporting on bowel function (e.g., frequency, consistency, urgency, incontinence) and QoL	Studies that do not report relevant outcomes for LARS management
Language	Published in English	Published in other languages
Species	Studies conducted in humans	Studies conducted on animals

Information Sources

The comprehensive literature survey was conducted from 8 July to 5 August 2024, utilizing multiple databases, including PubMed, MEDLINE, Cochrane Library (CENTRAL), ScienceDirect, Europe PMC, EBSCO Open Dissertations, Google Scholar, and the Clinical Trials registry [29]. The search strategy utilized concepts of keywords, Boolean operators, and medical subject headings (MeSH terms) to identify the pertinent studies (Table 2). The search focused on three main concepts: the condition of LARS, various management strategies for LARS, and the outcomes related to bowel function and QoL.

**Table 2:** Search Strategy EBSCO: Elton B. Stephens Company; LAR: low anterior resection; LARS: low anterior resection syndrome; MeSh: medical subject headings; PMC: PubMed Central; RCT: randomized clinical trials.

Search Strategy	Databases/Registers	Number of studies before and after filters	Filters applied
(("Low anterior resection syndrome" [Title/Abstract]) AND ("Management" [Title/Abstract] OR "treatment" [Title/Abstract] OR "Pharmacological treatment" [Title/Abstract] OR "medical management" [Title/Abstract] OR "Antidiarrheal" [Title/Abstract] OR "Bulking agents" [Title/Abstract] OR "pelvic floor exercise" [Title/Abstract] OR "Kegel exercises" [Title/Abstract] OR "Electrical stimulation" [Title/Abstract] OR "Rehabilitation" [Title/Abstract] OR "Surgical intervention" [Title/Abstract] OR "stoma formation" [Title/Abstract] OR "Pouch Reconstruction" [Title/Abstract]) AND ("Bowel function" [Title/Abstract] OR "Incontinence" [Title/Abstract] OR "quality of life" [Title/Abstract] OR "Complications" [Title/Abstract] OR "Adverse effects" [Title/Abstract]))	PubMed	233/18	Full text, Clinical Study, Clinical Trial, Observational Study, RCT, in the last 10 years, Humans, English, Adult: 19+ years.
("Low Anterior Resection Syndrome"[Mesh]) AND ("rehabilitation"[Subheading] OR "therapy"[Subheading] OR "Therapeutics"[Mesh] OR "Surgical Procedures, Operative"[Mesh]) AND ("Quality of Life"[Mesh] OR "complications"[Subheading] OR "adverse effects"[Subheading] OR "Defecation"[Mesh])	Medline	53/9	Full text, Clinical Study, Clinical Trial, Observational Study, RCT, in the last 10 years, Humans, English, MEDLINE
#1 (Low anterior resection syndrome):ti,ab,kw; #2 MeSH descriptor: [Low Anterior Resection Syndrome] explode all trees; #3 #1 OR #2 #4 ("Management" OR "treatment" OR "Pharmacological treatment" OR "medical management" OR "Antidiarrheal" OR "Bulking agents" OR "pelvic floor exercise" OR "Kegel exercises" OR "Electrical stimulation" OR "Rehabilitation" OR "Surgical intervention" OR "stoma formation" OR "Pouch Reconstruction"); #5 MeSH descriptor: [Therapeutics] explode all trees; #6 MeSH descriptor: [Rehabilitation] explode all trees; #7 #4 OR #4 OR #6 ; #8 "Improvement in bowel function" OR "Reduced Incontinence" OR "quality of life" OR "complications of management" OR "Adverse effects of management"; #9 #3 AND #7 AND #8	Cochrane Library	88/83	Cochrane trials Last 10 years English
Title, abstract, keywords: (“low anterior resection syndrome" OR "LARS") AND ("management" OR "treatment" OR "rehabilitation") AND ( "bowel function" OR "incontinence" OR "quality of life")	Science Direct	92/48	Time frame 2014-2024 English, Research articles. Subject areas: Medicine and Dentistry, Nursing and Health Professions
(("Low anterior resection syndrome" OR "Post Low anterior resection complication" OR "Post- surgery Low anterior resection complaints" OR "Low anterior resection /complication") AND ("Management" OR "treatment of LARS" OR "Pharmacological treatment" OR "medical management" OR "Antidiarrheal in LARS" OR "Bulking agents for LARS" OR "pelvic floor exercise" OR "Kegels exercise" OR "Electrical floor stimulation" OR "Rehabilitation" OR "Surgical intervention" OR "stoma formation" OR "Pouch Reconstruction") AND ("Improvement in bowel function" OR "Reduces Incontinence" OR "quality of life" OR "complications of management" OR "Adverse effects of management")) NOT ("review" OR "meta-analysis")	Europe PMC	78/47	Research articles. Link to full text. Full text in Europe PMC
(("low anterior resection" OR "LAR syndrome") AND ("bowel dysfunction" OR "fecal incontinence" OR urgency)) AND ("pelvic floor therapy" OR biofeedback OR "pharmacological treatment" OR "bulking agents") AND ("quality of life" OR "fecal incontinence" OR urgency OR frequency) AND (RCT OR "cohort study" OR "prospective study") NOT (review OR meta-analysis)	EBSCO Open Dissertations	17/ 8	Time frame 2014-2024
Low anterior resection syndrome OR Post Low anterior resection complication OR Post- surgery Low anterior resection complaints OR Low anterior resection /complication | Management OR treatment of LARS OR Pharmacological treatment OR medical management OR Antidiarrheal in LARS OR Bulking agents for LARS OR pelvic floor exercise OR Kegels exercise OR Electrical floor stimulation OR Rehabilitation OR Surgical intervention OR stoma formation OR Pouch Reconstruction | Adult (18 - 64) | Interventional, Observational studies | Studies with results | Outcome measure: Improvement in bowel function OR Reduces Incontinence OR quality of life OR complications of management OR Adverse effects of management	ClinicalTrails.gov	1 / 0	Adult (18 - 64) | Interventional, Observational studies | Studies with results
(Low anterior resection syndrome OR Post Low anterior resection complication OR Post- surgery Low anterior resection complaints OR Low anterior resection /complication) AND (Management OR treatment of LARS OR Pharmacological treatment OR medical management OR Antidiarrheal in LARS OR Bulking agents for LARS OR pelvic floor exercise OR Kegels exercise OR Electrical floor stimulation OR Rehabilitation OR Surgical intervention OR stoma formation OR Pouch Reconstruction ) AND (Improvement in bowel function OR Reduces Incontinence OR quality of life OR complications of management OR Adverse effects of management)	Google Scholar	3310/1620	From 2014-2024

Study Selection

The titles and abstracts of the studies obtained for eligibility in this review were independently examined by two reviewers. Articles deemed potentially relevant were also retrieved, provided that the full text was available. Any discrepancies between the reviewers were resolved through discussion or, if necessary, by consulting a third reviewer.

Data Extraction and Synthesis

After identifying the relevant studies, data were extracted to capture all essential information, including study characteristics, population demographics, intervention specifics, outcome measures, and follow-up durations. These results were systematically tabulated for clarity.

Quality Assessment

The quality of the included studies was evaluated using appropriate instruments. The Cochrane Risk of Bias Tool (ROB 2) was employed for randomized controlled trials (RCTs) [16], while the Newcastle-Ottawa Scale (NOS) was used for cohort studies [17].

Overview of Management Strategies

An objective overview of management strategies for LARS was developed based on the available evidence. A qualitative analysis of the pertinent study results was conducted and summarized. A p-value of <0.05 was considered indicative of statistical significance for the outcomes.

Results

**Figure 1:**
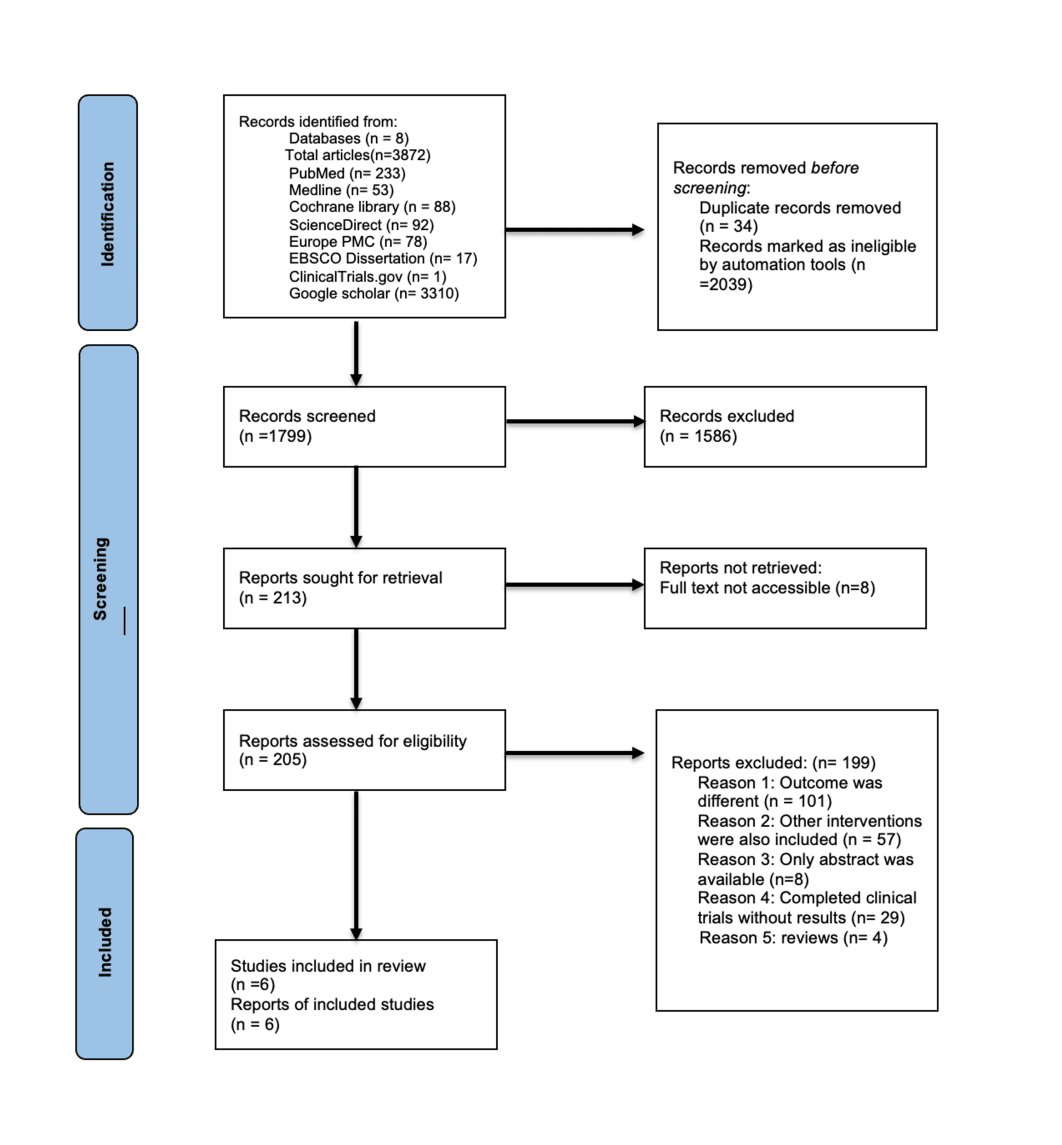
PRISMA Flow Diagram PRISMA: Preferred Reporting Items for Systematic Reviews and Meta-Analyses

A thorough search strategy produced 3,872 initial records from various databases. The Rayyan app® [18] was used to import these records, remove duplicates, and enable two independent reviewers to screen titles and abstracts. Following this initial screening, full texts were reviewed, resulting in a total of six eligible studies with full-text availability. Figure 1 outlines the methodology for the literature search and selection process.

The quality of the two observational studies was evaluated using the NOS [17]. Studies deemed acceptable for inclusion in the review achieved a score of at least seven out of nine points (equivalent to 77%). Both studies demonstrated good quality overall, with scores ranging from eight to nine. Herzberg et al. (2021) [8] received a score of eight, reflecting strong selection and outcome assessments, although it lacked a non-exposed cohort. Pieniowski et al. (2022) [13] achieved a score of nine, showcasing robust comparability and outcome measures, along with a well-defined non-exposed group. Overall, the studies exhibit a low risk of bias, with strengths in participant selection, comparability of cohorts, and reliable outcome assessments. The results suggest that these studies offer important insights into the impact of various interventions in surgical contexts, contributing to the understanding of patient outcomes following rectal surgeries (Table 3).

**Table 3:** Quality Assessment of Observational Studies: Newcastle-Ottawa Scale Scores Note: A maximum of four stars (*) could be awarded for selection, two for comparability, and three for outcome domains. Total score ranges from zero to nine.

Study by /Year of Publication	Selection	Comparability	Outcome	Overall
Herzberg et al., 2021 [8]	* * * *	*	* * *	8/Good quality
Pieniowski et al., 2022 [13]	* * * *	* *	* * *	9/Good quality

The four RCTs incorporated for this review were evaluated for bias risk utilizing the Cochrane RoB 2 (reference) and revealed varying levels of concern across different domains (Table 4).

**Table 4:** Risk of Bias Assessment of Randomized Controlled Trials: Cochrane Risk-of-Bias Tool (RoB 2) Note: RoB 2 domains: 1) Randomization process, 2) Deviations from intended interventions, 3) Missing outcome data, 4) Measurement of outcome, 5) Selection of reported result. +: low bias risk; -: high bias risk; !: some concerns about risk of bias.

Study by /Year of Publication	Domain 1	Domain 2	Domain 3	Domain 4	Domain 5	Overall
Ye et al., 2023 [19]	+	!	+	+	+	!
Marinello et al., 2024 [14]	+	!	+	!	+	!
Asnong et al., 2022 [20]	+	+	+	!	+	!
Rosen et al., 2019 [12]	+	+	+	+	+	+

Ye et al. (2023) [19] exhibited a low degree of prejudice in randomization and outcome measurement; however, their single-blind design raised some concerns regarding intervention delivery. Similarly, Marinello et al. (2024) [14] demonstrated robust randomization but faced potential unblinding issues and concerns about the specificity of the LARS score used for outcome measurement, resulting in an overall assessment of certain concerns. Asnong et al. (2022) [20] showed low risks in randomization and outcome reporting but utilized a non-validated stool questionnaire for secondary outcomes, introducing measurement bias. Lastly, Rosen et al. (2019) [12] presented low risks in the randomization and selection of reported results but raised concerns regarding missing outcome data and blinding. Collectively, while these studies provide valuable insights, the identified biases necessitate a cautious interpretation of their findings, emphasizing the importance of rigorous methodological standards in clinical research.

Our systematic review identified that all six incorporated studies demonstrated acceptable quality levels. While some concerns were noted in various domains of the bias risk assessments for both observational and RCT studies, these did not preclude their inclusion in the conclusive evaluation.

Characteristics of Included Studies

This systematic review includes studies employing various designs, including prospective cohorts, RCTs, and retrospective cohorts, to evaluate patient outcomes related to LARS. A total of 794 adult participants were included, with sample sizes ranging from 37 to 430 in individual studies. The overall gender distribution across the studies shows a higher representation of males compared to females in the pooled population.

The main subject of these studies encompasses the evaluation of QoL, patient-reported outcomes, and specific LARS symptoms. Key metrics assessed include global health status (GHS), LARS scores, bowel function, and the effectiveness of various educational and therapeutic interventions, including pelvic floor rehabilitation and sacral neuromodulation. Table 5 summarizes the included studies, detailing their design, sample size, participant demographics, objectives, and key findings.

**Table 5:** Summary of Included Studies. 3D: Three-dimensional; CG: Control Group; EG: Experimental Group; FU: Follow-up; GHS: Global health status; IG: Intervention Group; IPG: Implantable pulse generator; LAR: Low Anterior Resection; LARS: Low Anterior Resection Syndrome; LARSS: Low Anterior Resection Syndrome Score; NRS: Numerical Rating Scale; PFMT: Pelvic floor muscle training; QoL: Quality of Life; RC: Rectal cancer; RCT: Randomized controlled trial; SD: Standard Deviation; SF-36: The 36-Item Short Form Health Survey questionnaire; SF-12: 12-Item Short Form Health Survey; TAI: Transanal irrigation; TME: Total mesorectal excision.

Author/Year	Study Design	Sample Size	Age/Sex	Aim of Study	Key Findings
Herzberg et al., 2021 [8]	Prospective Cohort	78	Mean Age: 65.64 ± 12.24 years Gender: Males; 57.7% Females; 42.3%	To explore the outcomes reported by the patient’s following LAR with primary reconstruction and defunctioning ileostomy utilizing a standardized perioperative fail-safe protocol.	The mean GHS was 67.95 points, indicating generally favorable QoL. 61.5% of the cohort experienced Major LARS. There were no cases of anastomotic leakage reported, translating to positive effects of defunctioning ileostomy on LARS. Longer operative times were associated with major LARS (p <0.05), suggesting prolonged surgeries and complications during surgeries contribute to major LARS and affects bowel function.
Ye et al., 2023 [19]	RCT	99	1) The CG (n=31) Mean Age: 57.61 ± 7.36 Gender: Males: 18 (58.10 %) Females: 13 (41.90%) 2) EG I (n=32) Age: 57.03 ± 7.51 Gender: Males: 20 (62.50 %) Females: 12 (37.50 %) 3) EG II ( n=31) Age: 56.68 ± 6.89 Gender: Males:19 (61.30 %) Females: 12 (38.70 %)	To evaluate the effect of 3D animation combined with the teach-back health education on the recovery of LARS patients. Training Methods: CG: One-to-one verbal guidance on pelvic floor muscle training. EG I: One-to-one guidance with 3D animation. EG II: 3D animation combined with teach-back method.	CG: Lower training content completion; total LARSS scores: Preoperative: 33.03 ± 3.52, 1 month: 31.58 ± 4.00, 3 months: 30.87 ± 3.77. EG I: Improved training content completion; total LARSS scores: Preoperative: 32.88 ± 4.23, 1 month: 30.03 ± 3.41, 3 months: 27.50 ± 3.60 (lower than CG but higher than EG II). EG II: Highest training content completion; total LARSS scores: Preoperative: 32.71 ± 4.23, 1 month: 29.65 ± 3.59, 3 months: 25.48 ± 2.16. EG II outperformed CG and EG I in defecation urgency, frequency, and loose stool incontinence at various time points (14 days, 1 month, and 3 months post-operation) (p <0.05).
Pieniowski et al., 2022 [13]	Retrospective Cohort	430	1)No Stoma Group: Mean Age: 67 years (SD: 12) Gender: Males (46%), Females (54%) 2) Defunctioning Stoma Group (n=350): Mean Age: 64 years (SD: 9) Gender: Males (59%), Females (41%)	To investigate the association between a defunctioning stoma (used as a preventive measure) and long-term bowel function, specifically major LARS.	Defunctioning stoma (temporary ileostomy) associated with higher LARS scores. Major LARS occurs in 57% of patients with a stoma. No clear link between stoma reversal time and QoL. Mean LARS score: 29 (stoma) vs. 21 (no stoma)
Rosen et al., 2019 [12]	RCT	37	1)TAI Group: Mean Age: 58.5 years Sex Ratio: Males: 12 Females: 6 2) CG: Mean Age: 58 years Sex Ratio: Males: 5 Females: 14	To evaluate the effect of prophylactic TAI in preventing symptoms of LARS after rectal resection.	Significant improvement in LARS scores (median 16 vs. 32 at 1 month; P = 0.044; median 9 vs. 31 at 3 months; P = 0.001). Lower maximum stool episodes per day (3 vs. 7 at 1 month; 3 vs. 5 at 3 months). Improved Wexner scores (median 2 vs. 6 at 3 months; P = 0.046). QoL via the SF‐36®️ questionnaire failed to show any differences between the groups.
Marinello et al., 2024 [14]	RCT	46	IPG Implantation: 1) No: Median Age: 59.0 years Sex: Females (27.3%), Males (72.7%) 2) Yes: Median Age: 64.0 years Male: (68.6%) Female: (31.4%)	To assess the impact of sacral neuromodulation on LARS symptoms, using validated assessment tools such as the LARS score and QoL questionnaires.	78% of patients had a ≥50% reduction in LARS score after the intervention of sacral neuromodulation. Baseline mean LARS score: 37.94; mean reduction: 15.51 (59.12%) after testing. At 6 months: mean reduction –6.2 (p < 0.001); at 12 months: –6.97 (p < 0.001). QoL improvements included: GHS score increased from 0.55 to 0.75 (p < 0.01), fatigue reduction (p < 0.05), pain reduction (p < 0.01), and decreased diarrhea episodes (p < 0.01). Decreased urgency episodes (p < 0.001), improved sensation of bowel emptying, and better stool/gas discrimination.
Asnong et al., 2022 [20]	RCT	104	1) IG: Mean Age: 58.8 years Male: (72.0%) Female:(28.0%) 2) CG: Mean Age: 57.1 years (SD: 10.9) Male: (64.8%) Female:(35.2%)	To investigate the effectiveness of PFMT on LARS in patients after TME for rectal cancer.	Improvement in LARS scores at 4 months: 38.3% (PFMT) vs. 19.6% (control) (p=0.0415). At 6 months: 47.8% (PFMT) vs. 21.3% (control) (p=0.0091). Continuous LARS scores decreased significantly at 4 months (P=0.0496). Reduced bowel movements (p=0.0277), solid stool leakage (day: P=0.0241; night: P=0.0496), and clustering (P=0.0369) observed at 4 months. No significant differences in QoL scores (NRS and SF-12).

Discussion

This review offers an extensive overview of the current landscape in the management of LARS, highlighting the persistent limitations and challenges within this field. The findings underscore the complexity and heterogeneity inherent in LARS management, advocating for multifaceted and individualized treatment approaches. 

Pelvic Floor Rehabilitation

Rehabilitation, encompassing training of pelvic floor muscles (PFMT) and feedback has emerged as a promising intervention for improving anorectal function, particularly in reducing urgency and incontinence. These modalities focus on strengthening pelvic floor muscles, enhancing the coordination of the defecation reflex, and retraining anorectal muscles, which are often disrupted following LAR.

Two studies: Ye et al. [19] and Asnong et al. [20] elucidate the positive effects of pelvic floor rehabilitation. To demonstrate the positive impact of pelvic floor rehabilitation on LARS syndrome, Ye et al. conducted an RCT study that included a control group (CG) and two experimental groups: Experimental Group I (EGI), which utilized 3D methods with teach-back (a technique where participants explain back what they have learned to ensure understanding), and Experimental Group II (EGII), which utilized 3D methods without teach-back. The CG received only nurse-led one-to-one verbal education [19]. All three groups demonstrated a reduction in LARS scores. Notably, the EGII group exhibited the most significant improvements. The LARS score for EGII was significantly lower at both one and three months, recorded at 29.65 ± 3.59 and 25.48 ± 2.16, respectively, compared to the pre-operative score of 32.71 ± 4.23 (p <0.05). Furthermore, EGII showed enhancements in LARS scores related to stool frequency, urgency, and reduced incontinence. These findings underscore the effectiveness of a multifaceted approach to pelvic floor rehabilitation, resulting in substantial improvements in LARS scores and overall QoL [19].

Similarly, Ansnong et al. developed a multicentre, single-blind, randomized controlled trial (RCT) comparing pelvic floor muscle training (PFMT) with no PFMT, which demonstrated notable improvements in LARS at four months (38.3% vs. 19.6%; P = 0.0415) and six months post-surgery (47.8% vs. 21.3%; P = 0.0091). However, these benefits diminished at the 12-month mark [20]. Given the rapid recovery of symptoms associated with PFMT, this study advocates for its early implementation as an intervention [20].

This research highlights that pelvic floor rehabilitation serves as a fundamental treatment option, primarily because it is non-invasive. Patients undergoing rehabilitation reported significant enhancements in QoL, particularly when the urgency and tenesmus were resolved, which led to improved comfort in patients' lives [19]. Nonetheless, as with functional outcomes, the effectiveness and long-term impact on QoL vary among patients and are closely related to the improvement in the LARS score that Ye et al. have described at various times during the rehabilitation program [19]. Consequently, the long-term sustainability of pelvic floor rehabilitation remains uncertain, with success depending on the severity of LARS symptoms and the timing of initiation [20].

Despite these promising results, the long-term sustainability of PFMT remains uncertain. While patients report improvements in QoL, variability in individual responses indicates that factors such as the severity of LARS symptoms and the timing of the intervention play critical roles in treatment success. Thus, a tailored approach to pelvic floor rehabilitation is essential, focusing on patient motivation and specific symptom management.

Role of Surgery in Managing LARS

Surgery plays a crucial role in managing LARS, emphasizing both prevention and definitive treatment. Key factors include the type of anastomosis and the use of defunctioning stomas. Herzberg et al. demonstrated that primary reconstruction via end-to-end anastomosis when standardized pathways are followed, reduces the risk of LARS by preventing anastomotic leaks [8]. However, longer operative times are associated with an increased risk of major LARS (p < 0.05) [8]. Emerging surgical techniques, such as transanal total mesorectal excision (TaTME), have shown favorable outcomes [21]. Additionally, short stump and high anastomosis pull-through (SHiP) procedures exhibit low incidences of LARS, supporting the avoidance of routine stoma use during primary surgery [22].

Pieniowski et al. found that defunctioning stomas correlate with a higher incidence of major LARS, and while stomas may provide short-term protection, prolonged use can hinder bowel adaptation and worsen long-term function [13]. Our systematic review found no link between the timing of stoma reversal and future bowel dysfunction, aligning with Vogel et al.'s meta-analysis, which recommended early closure of defunctioning ileostomies to mitigate adverse effects on bowel function [23]. For refractory LARS, surgical intervention is often necessary, with a permanent colostomy occurring in about 2-3% of patients [24]. Garfinkle et al. noted that a stoma may be a last resort for managing refractory cases, although surgery carries significant risks and psychological implications, including depression and social isolation [11,24]. With appropriate support, patients can improve their QoL [25]. Stoma care nurses play a vital role in this process, offering education, dietary advice, and monitoring symptoms to facilitate smoother postoperative transitions [26].

Another surgical option is sacral neuromodulation, which involves implanting a neurostimulation device. The SANLARS trial by Marinello et al. showed that 78% of patients experienced a significant reduction in LARS scores and improved QoL metrics (p <0.001) [14]. However, this approach is most effective for patients with incontinence rather than evacuation issues, necessitating careful patient selection [11].

Transanal Irrigation

Transanal irrigation (TAI) has emerged as an innovative intervention for managing LARS. This minimally invasive procedure can be performed at the bedside by the patients themselves, utilizing saline to flush the colon [27]. Our review indicates that TAI significantly enhances bowel function, with patients reporting notable improvements in stool frequency both during the day and at night, at one and three months. Furthermore, there were significant reductions in LARS and Wexner scores in the TAI group (p <0.05) [12].

Introducing TAI early in the management of LARS, especially for patients at risk, can optimize outcomes [11]. Although rare, there is a risk of rectal perforation, estimated at 6 per million [28], warranting further investigation. The long-term safety and efficacy of TAI remain uncertain, but ongoing trials in Denmark by Christensen et al. comparing glycerine suppositories to TAI may provide valuable insights [28].

While TAI is a relatively new treatment for LARS, the results are promising. Patients report reduced stool frequency and improved bowel function, particularly when the therapy is introduced early. Although the risk of rectal perforation is low, it remains a significant concern, and ongoing research is expected to shed more light on this intervention.

Comparisons Across Interventions

In comparing therapeutic approaches, this review highlights that no single modality is superior; rather, each offers distinct benefits with varying effectiveness. It emphasizes the importance of a multimodal, tailored, nuanced, and patient-centered management approach that balances efficacy, safety, and QoL.

For patients with mild to moderate LARS symptoms, pelvic floor rehabilitation can yield substantial benefits, particularly for motivated patients willing to engage in long-term therapy; however, it necessitates ongoing participation to maintain efficacy. Transanal irrigation (TAI) is another effective and minimally invasive treatment option that significantly improves bowel function, reduces stool frequency, and lowers LARS and Wexner scores when introduced early in therapy. Despite its benefits, the procedure carries a rare but serious risk of rectal perforation, with long-term safety still under investigation.

In contrast, surgical options are typically considered when other treatments have been exhausted and are best suited for cases of severe, refractory LARS. While these surgical interventions are highly effective in severe cases, they carry significant risks and psychological impacts. Integrating these modalities, with or without combining pelvic floor rehabilitation, followed by surgical options in refractory LARS, provides a more comprehensive strategy for LARS management. Addressing the chronic nature and complex challenges of LARS requires long-term follow-up, continuous patient support, ongoing research, and a tailored approach.

Strengths and Limitations of Included Studies

The included studies demonstrated several notable strengths that enhance the credibility of our findings. They showcased a diverse array of research designs, employing methodologies such as RCTs and cohort studies, which provide a comprehensive perspective on the management of LARS. Furthermore, many of these studies featured large sample sizes, which significantly enhance the statistical power of their findings and can improve generalizability to broader populations. Longitudinal follow-up was another notable strength, as several studies tracked participants over extended periods, facilitating the evaluation of long-term effects and outcomes related to LARS management. Additionally, the studies generally adhered to rigorous methodological standards, following established guidelines that minimize bias and ensure the reliability of the results.

Despite these strengths, the included studies exhibited significant limitations that affect generalisability. While large sample sizes can enhance the applicability of findings, heterogeneity among the studies, stemming from variations in participant characteristics, analytical methods, specifics of interventions, and evaluation metrics, complicates the ability to draw definitive, overarching conclusions. This variability lowers the generalisability of the findings, as results may not apply uniformly across all populations or clinical contexts. Moreover, the generalisability of the findings is further constrained by variations in cultural, socioeconomic, and clinical contexts. Bias risk was also present in some studies, stemming from factors such as selection bias, measurement bias, and confounding variables. Lastly, some studies suffered from shortcomings in reporting, lacking sufficient detail in their methods, results, or limitations, which hindered the assessment of their overall quality and relevance.

Assessment of the Systematic Review

The systematic review itself exhibited several strengths. It likely utilized a comprehensive search strategy to retrieve pertinent studies, thereby minimizing the risk of overlooking important evidence. The eligibility criteria were clearly defined, ensuring consistency and transparency throughout the review process. Additionally, the review utilized validated assessment tools to evaluate the bias risk of the suitable studies, thereby offering substantive insights into the quality of the evidence presented. Transparent reporting was another hallmark of the review, as it clearly described the methods used, the results obtained, and the conclusions drawn, enabling reproducibility and critical appraisal.

Nevertheless, the review has its limitations. There is a potential for publication bias, given that studies with favorable or statistically significant outcomes tend to have a higher probability of publication, in contrast to those with negative or non-significant findings. Subjectivity in assessment also poses a challenge; despite efforts to minimize this, variations in the interpretation and evaluation of the included studies could still occur. Furthermore, the review may have faced limitations due to the limited availability of full-text articles, which could restrict access to complete data or methodological information for some identified studies.

Future Research Directions

Building upon the strengths and limitations identified in the comprehensive studies, alongside the overall assessment of the systematic review, several key research directions emerge. First and foremost, addressing the gaps in current evidence is crucial. One significant area for future research is the need for parallel comparisons of variable interventions for LARS. Studies that directly compare various treatment modalities, such as pelvic floor muscle training versus transanal irrigation versus surgical options, would yield more definitive evidence regarding the relative effectiveness of these approaches.

Future research should prioritize longitudinal studies to evaluate the long-term outcomes associated with LARS management strategies. Such studies are crucial for understanding how the effects of interventions may change over time and how symptoms might evolve. By focusing on these aspects, researchers can gain deeper insights into the sustainability of treatment benefits and the long-term needs of patients, ultimately informing more effective management strategies for LARS. Such research would enhance our understanding of the long-lasting effects if followed up over an extended period. Another important avenue for exploration is the cost-effectiveness of different LARS interventions. While some studies have begun to assess the economic implications of various treatments, there remains a significant need for comprehensive evaluations. By systematically analyzing the cost-effectiveness of these interventions, future research can inform clinical practice and healthcare policy, ultimately identifying the most efficient and effective strategies in terms of clinical outcomes and resource utilization.

Furthermore, investigating patient-reported outcomes is vital to comprehensively understanding the influence of LARS on an individual’s QoL. Employing validated outcomes would facilitate the evaluation of the physical, psychological, and social consequences of LARS, thereby enriching the body of evidence surrounding patient experiences. To explore how patient-specific elements, such as age, gender, comorbidities, and surgical technique, affect LARS outcomes and the effectiveness of different interventions.

Finally, as new therapies for LARS continue to emerge, future studies should rigorously evaluate their efficacy and safety in comparison with established treatments. By pursuing these research directions, future investigations can significantly enhance the care provided to patients with LARS and offer more definitive evidence to guide clinical practice and decision-making.

## Conclusions

This systematic review assessed current management strategies for LARS, highlighting various interventions such as pelvic floor rehabilitation, surgical options, and minimally invasive techniques like transanal irrigation. While some studies indicate positive effects on bowel function and quality of life, the effectiveness of these interventions varies significantly. The optimal management strategy for LARS remains uncertain, influenced by patient characteristics, symptom severity, and individual preferences.

Employing a systematic, stepwise approach-from non-invasive measures to surgical intervention when necessary effectively mitigates the debilitating effects of LARS. Future research is crucial to address existing limitations and gaps in the evidence. Head-to-head comparisons of interventions, longitudinal studies on long-term outcomes, and cost-effectiveness analyses are essential for informed clinical decision-making. Additionally, thorough assessments of patient-reported outcomes and exploration of patient-specific factors and emerging therapies will enhance care for LARS patients and clarify optimal management strategies.
